# The PHA4GE Microbial Data-Sharing Accord: establishing baseline consensus microbial data-sharing norms to facilitate cross-sectoral collaboration

**DOI:** 10.1136/bmjgh-2024-016474

**Published:** 2024-10-30

**Authors:** Emma J Griffiths, Peter van Heusden, Tsaone Tamuhla, Eddie T Lulamba, Anja Bedeker, Michelle Nichols, Alan Christoffels, Nicki Tiffin, On behalf of the Public Health Alliance for Genomic Epidemiology (PHA4GE) Ethics and Data Sharing Working Group

**Affiliations:** 1 Simon Fraser University Faculty of Health Sciences, Burnaby, Ottawa, Canada; 2 South African Medical Research Council Bioinformatics Unit, South African National Bioinformatics Institute, University of the Western Cape, Bellville, South Africa; 3 College of Nursing, Medical University of South Carolina, Charleston, South Carolina, USA; 4 Africa CDC, African Union, Addis Ababa, Ethiopia

**Keywords:** Medical microbiology

## Abstract

Microbial data sharing underlies evidence-based microbial research, as well as pathogen surveillance and analysis essential to public health. While the need for data sharing was highlighted during the SARS-CoV-2 pandemic, some concerns regarding secondary data use have also surfaced. Although general guidelines are available for data sharing, we note the absence of a set of established, universal, unambiguous and accessible principles to guide the secondary use of microbial data. Here, we propose the Public Health Alliance for Genomic Epidemiology (PHA4GE) Microbial Data-Sharing Accord to consolidate consensus norms and accepted practices for the secondary use of microbial data. The Accord provides a set of seven simple, baseline principles to address key concerns that may arise for researchers providing microbial datasets for secondary use and to guide responsible use by data users. By providing clear rules for secondary use of microbial data, the Accord can increase confidence in sharing by data providers and protect against data mis-use during secondary analyses.

**SUMMARY BOX**

- Secondary use of the ever-increasing volumes of microbial and pathogen data being generated through research and surveillance activities can maximise benefits from their use.
- Currently, consensus terms and conditions for re-using microbial data are unclear, and there is not a well-recognised or generally accepted etiquette, or set of baseline rules, for their re-use.
- The Ethics and Data Sharing Working Group of the Public Health Alliance for Genetic Epidemiology (PHA4GE), in consultation with stakeholder groups, is presenting the PHA4GE Microbial Data-Sharing Accord, a document describing a set of baseline, common decency principles to govern the re-use of microbial data.
- The Accord is intended to act as a consensus baseline of expected behaviours for microbial data re-use, setting a common standard on which microbial data-sharing arrangements may be based.
- The Accord should not replace specific legal agreements or memoranda between sharing partners but rather provide a common starting place for agreeing data-sharing behaviours that are unambiguous, are clearly stated and reflect a consensus from a wide range of stakeholders.

## Introduction

 The use and re-use of data from diverse sources underpins evidence-based healthcare, highlighting the importance of data sharing to maximise benefits for health systems and their healthcare clients globally. Principles and frameworks to aid data sharing have been developed, for example the FAIR principles,^1^ and the WHO Policy on Data Sharing in the Context of Public Health Emergencies.^2^ The enormous value of sharing microbial data during a global infectious disease crisis was unequivocally demonstrated during the SARS-CoV-2 pandemic,^3^ with concomitant recognition of the need for guidelines and recommendations on data sharing in the context of pandemic response.^4^ Many of these guidelines and policies focus on the availability and useability of data, for example the FAIR principles promote Findability, Accessibility, Interoperability and Reusability of data, to ensure that secondary users are able to find, access and seamlessly integrate and analyse datasets that have been generated in previous research by other parties; but there are few existing data-sharing frameworks that focus on issues of equity or ethics that impact the research participants or original generators from whom the data are received. The African Academy of Sciences Recommendations for Data and Biospecimen Governance in Africa is one of the few such policies that focus on participant protection and equitable practices.^5^

Sharing pathogen data, including epidemiological data, pathogen genome sequence data, proteomic data and pathogen-host contextual and environmental data, can promote research by enabling enhanced meta-analyses to better understand phylogenetics and evolution, pathogenicity, antimicrobial resistance and other pathogen characteristics. In addition, these data are highly relevant for effective surveillance and outbreak tracking and to design early warning systems for potential and emerging infectious disease threats.

Microbial and pathogen data may have diverse origins, for example they may be generated by public health laboratories in the course of providing surveillance and diagnostics as a public health service, or they could be the output of bespoke microbial research undertaken by academic researchers. Public health laboratories generate pathogen data in the course of fulfilling their mandate for disease surveillance, outbreak response and public health research. They conduct diagnostic testing, synthesise epidemiological data and develop strategies to prevent and control the spread of infectious diseases, and they collaborate with other public health agencies, healthcare providers and researchers to ensure timely and accurate information for effective public health interventions. A similar role for veterinary and agricultural pathogen diagnosis and surveillance is also fulfilled by governmental agencies and institutions responsible for animal health and food safety, whilst recognising the need for collaborative approaches to zoonotic pathogens; environmental protection agencies, water resource management authorities and agencies managing natural resources may also contribute to monitoring and research. Academic researchers generate microbial data from their research programmes exploring microbial biology, evolution and epidemiology for pathogenic, opportunistic and non-pathogenic microbes, for those with host organisms and for those found independently in the environment.

Coordination among many stakeholders and data generators is essential for assessing environmental risks, detecting emerging threats, and implementing strategies to mitigate public health and environmental impacts; and this requires trusted avenues for rapid sharing of microbial data to ensure a timely, evidence-informed response. Such data sharing also facilitates secondary data use, however, where the data are used for purposes beyond the original reason for data collection - for example when researchers use data generated by public health agencies for research that is different to the original use for public health diagnostics and surveillance. Recognising the different deliverables and outcomes for the diverse stakeholders who may generate, access and analyse microbial data underscores the need to articulate terms and conditions of data sharing very clearly, as they may often be misconstrued depending on the local context, objectives and data-use norms for each type of stakeholder. As described below, the understanding of what conditions are in place when sharing microbial data for secondary analyses can be unclear where there are no specific data-sharing agreements in place - for example where microbial data have been uploaded to a centralised data repository beyond the oversight of the data generator in terms of types of onward use.

Onward sharing of different types of data generated from human research participants and healthcare clients has received extensive and appropriate attention, and is strongly regulated and monitored due to demonstrable potential for harms to participants through breach of privacy and impingement on human rights. Protections are enshrined in legislation such as Health, Data Protection, and Protection of Privacy Acts, and the importance of upholding clearly articulated ethical principles when undertaking research involving human participants is well-established. Informed consent from participants is mandatory for any research where risks or potential for participant re-identification exists.^6^,^8^ When working with microbial and pathogen data, the ethical and privacy implications for host data are less clearly delineated, even when the host is a human being. Working with data from isolated microbes has minimal risks for humans, but when working with pathogen data, analyses are often incomplete without the inclusion of health metrics from the infected individual in order to assess the clinical impact of the pathogen. For surveillance and analysis of pathogen distribution, geospatial coordinates may be identifying data for households and communities; and samples for analysis are often contained within the blood or tissues collected from individuals which may subsequently also generate contaminating human DNA sequence when processed. Without unequivocal guidelines and requirements in place, these blurred boundaries about risk to individuals arising from data sharing can sometimes lead to inappropriate secondary analyses that compromise the participants in the research, and by extension also those who are responsible for the primary data collection.

Open data sharing of all pathogen data, including sequence data and sample meta-data, has been encouraged by many—especially researchers in the global North working in well-resourced environments.^9^ Others, predominantly in the global South, have raised concerns about the potential for exploitation, the failure to receive recognition for their contributions or to be included in downstream data processing, and the inequities and neo-colonial practices that dominate resource and benefit sharing with the global South—even where the data they generated are shared openly to support the development of those resources.^10^,^13^ This inequity was starkly highlighted in the delayed and limited availability of COVID-19 vaccines in the global South, even where countries had played a contributory role in generating sequence data and running vaccine clinical trials.^14 15^

Additional challenges have arisen around secondary use of data and subsequent research outcomes, such as a failure to recognise or acknowledge the data generators thus compromising the sustainability of their research, unauthorised re-use of datasets which may be coupled with inappropriate or ethically unsound onward use of data, insensitive or stigmatising reporting of identified communities or individuals^16 17^, and the potential for negatively impacting public health messaging and delivery through publication of inaccurate, contradictory or confusing secondary academic analysis of public health data. These challenges highlight that when data are released as Open data, this does not always mean that all uses of the data are appropriate, and with the rise in sharing of public health surveillance data during the pandemic, these challenges have become more acute. While there are some international recommendations for data sharing, for example the WHO’s data-sharing recommendations,^18^ these principles are subjective and non-specific; and there are no rules of ‘etiquette’ for taking and using data collected or generated by others. This makes it difficult to operationalise such principles and put them into concrete practice. Currently, norms for microbial and pathogen data sharing are implicit and are context-specific, making sharing across stakeholder domains loaded with the potential for misunderstandings and data misuse.^19 20^

With the rise in sharing of public health surveillance data during the SARS-CoV-2 pandemic, these challenges have become more acute, and while there are some international recommendations for data sharing from organisations such as the WHO,^18^ these are subjective principles, and there are no standardised rules, or ‘etiquette’, for taking and using others’ data. We propose the creation of a consensus document that can act as a **Data-Sharing Accord for Microbial Data**, with the understanding that ‘microbial data’ include data from pathogens, opportunistic pathogens and non-pathogenic microbes. This Accord aims to provide generally accepted, practical ‘rule-of-thumb’ elements for data users to observe when conducting secondary analyses with data generated by others, helping to operationalise generalised principles into simple unequivocal practice. We propose that this Accord should specifically govern the **secondary use** of microbial data and should be guided by a ‘common decency’ and ‘common sense’ approach that is applicable for both openly available data as well as private datasets governed by additional bespoke data-sharing agreements. It should recognise that microbial data are often generated by public health laboratories with a vested interest in research and research output but potentially limited capacity to engage directly with research, while recognising that the secondary use of data can also create risks for public health service laboratories. We anticipate that such a document can provide a simple and unequivocal way for data providers to advertise their expectations from data consumers—for example, with a statement such as ‘*These data are available for secondary analysis according to the principles of the PHA4GE Microbial Data Sharing Accord’*.

### Stakeholder engagement

The PHA4GE Ethics and Data-Sharing Working Group conceptualised the first draft of a short document that describes some fundamental principles for data sharing that can be used as a baseline set of understood norms for sharing microbial and pathogen data, and that is intended to be generally acceptable to the microbial and pathogen data-generating and data-using public health and research communities. This first draft was built on the combined experience and expertise of members of the group and the wider PHA4GE community and was then introduced to groups of stakeholders at The Global Microbial Identifier meeting (https://gmi13.org/) in Vancouver in September 2023), at the PHA4GE meeting (www.pha4ge.org) in Cape Town in October 2023, and at the Wellcome Connecting Science AMR Symposium in Johannesburg in March 2024 (https://coursesandconferences.wellcomeconnectingscience.org/event/genomics-for-antimicrobial-resistance-surveillance-and-one-health-20240315/). Feedback from engagement sessions was incorporated into finalising this first draft.

### Elements of the PHA4GA Data-Sharing Accord

The following areas were identified as those in which misunderstandings and misuse through secondary analysis most commonly occur, and we propose a clause, presented as a baseline rule, that articulates accepted best practice for each of these. Our intention is that it is possible for data generators to advertise the terms of use for secondary analysis with a proviso for exclusion or modification of certain clauses, for example ‘*These data are available for secondary analysis according to the principles of the PHA4GE Microbial Data Sharing Accord v1.0, with the exception of Clause 7: Opportunity for Collaboration, which is not required for the use of these data’*. In other words, data generators are not obliged to subscribe to all the clauses exactly as listed, and may select some to opt out of according to their own requirements. In addition, it is not our intention that the PHA4GE Data-Sharing Accord would replace the Data-Sharing Agreements currently in use, but instead would provide a baseline set of conditions for the secondary use of data that are shared openly, through existing access-controlled platforms, and/or are presented through publications or other media. For example, the statement above may also be included in the Data Availability section of a peer-reviewed article in a scientific journal. We describe below the rationale underlying each of the clauses, as they are presented in Box 1: The PHA4GE Microbial Data-Sharing Accord. The current version of the Accord (v1.0) is included as online supplemental file 1, and in the future the most recent version may also be found at: https://tinyurl.com/Microbial-DataSharing-Accord.

Box 1 **The PHA4GE microbial Data-Sharing Accord (version 1.0)**

**Definitions**

**Data generators:** The individuals and institutions that generated and/or collected the microbial data.

**Data consumers:** The individuals and institutions using microbial data for analysis.

**Secondary data use:** The use of pre-existing data, that were originally collected for a different purpose, to address new research questions or generate additional insights without conducting new primary data collection.

**Introduction**

**Aim:** The Accord aims to establish and delineate a set of baseline consensus norms for the sharing of both openly available and private microbial genomic datasets. The Accord aims to provide a single reference document that can provide a common set of standards for data producers and consumers regarding how secondary use of microbial genomic data will be conducted by those adhering to the principles of the Accord.

**Scope:** The Accord presents a consensus standard for ways of using data for secondary analyses that is clearly articulated in one place and is unambiguous. Whilst the Accord allows for additions and amendments for individual data-sharing scenarios, it provides a central baseline agreement underpinned by a common consensus understanding of how data may be used for further analyses.

**Benefits:** The Accord alleviates the requirement to continually recreate standardised clauses in every new data-sharing agreement. It provides a commonly understood and accepted baseline agreement for the use of shared microbial genomic data, which can be referred to simply as, for example, ‘*sharing according to the PHA4GE Microbial Data Sharing Accord’*.

**Clauses Regarding Secondary Use of Microbial Data**

1. Attribution

The Methods section of any type of publication using the data will explicitly describe the source of data, with links/citation where appropriate.

The Acknowledgements section of any type of publication using the data will explicitly describe the source of data, with links/citation where appropriate.

- Where there are very many data generators they may be listed in a supplementary table for a publication, or an online accessible list, for example a webpage or github repo, for oral presentations and other media. In these cases, if feasible, a publication may include a list of contributing institutions.

1. Overview of outputs prior to their publication

An email or alert will be sent to data generators with a confidential copy of a publication to review prior to publication.

A standard 2 week window will be provided for data generators to review the publication and raise any **serious concerns** that create a real and substantial risk for the data generators. No response within the 2 week window equates to no concerns raised, and only concerns about serious consequences should be raised.

- The duration of this review window may be increased by data generators but should not be decreased without very good cause.

1. Onward sharing of data

There will be no onward sharing of data to third parties unless there is explicit documented agreement from the data generator for onward sharing, or the data are already available for use as unrestricted open data.

1. Host and phenotype data

For human hosts, clinical data attached to sequence data will always be anonymised and de-identified such that they cannot be re-linked to individuals under any circumstances.

Genomic data will always be cleaned of contaminating human sequence by data generators prior to sharing, unless specific agreements and ethical clearance is in place for the onward use of these data.

For other species, genomic data will always be cleaned of contaminating host sequence (eg, animal and plant sequence data) to ensure compliance with trade and national resource laws and treaties.

1. Geospatial data

Heat maps will be used with aggregate data areas to plot geospatial data, to ensure individuals or specific communities cannot be identified.

- Individual sample geocoordinates representing humans infected with pathogens will not be presented as dots on maps, in order to prevent re-identification and/or stigma for individuals and communities affected by infectious diseases. Heat maps will ensure that counts per aggregate data area and the area covered are sufficiently high that they cannot be used to infer the identity of individuals, households or specific communities.

1. Intellectual property (IP)

IP does not transfer to the data user unless specifically and explicitly agreed by the data generator and supported by valid IP transfer agreement documentation.

- Unless there is a valid written agreement documenting a different arrangement, IP remains with the data generator.

1. Opportunity for collaboration

A reasonable attempt must be made by the data consumer to collaborate with the data generator where feasible, except where the data generator has provided an explicit waiver of this requirement.

#### Attribution

This clause aims to address the concern that data generators need acknowledgement and recognition when their data are re-used, to facilitate their justification for future funding, sustainability and ability to demonstrate the value of their work. This clause seeks to ensure the recognition of the source of the data and the use of appropriate links and citations in publications in both the Methods and Acknowledgements sections of publications.

#### Overview of outputs prior to publication

This clause aims to address the concern that secondary data analyses that are published by other parties may be damaging to public health programmes and messaging, or that mis-interpretations of the data may be misleading. This clause recognises that data generators may be at risk from publication of secondary analyses, and may have insights about the source of the data that could inform interpretation of subsequent analyses. This clause also recognises that publication of secondary analyses should not be held up indefinitely by data generators, and thus includes the proviso the data generators must be timely in their review of proposed publications otherwise they revoke this right automatically.

#### Onward sharing of data

This clause sets as default that those receiving the data for secondary analysis may not subsequently pass that dataset onward to other users for further analyses. This clause addresses the concern that datasets may become corrupted or modified in a way that renders them inaccurate, if copies are passed on from user to user. The preference is for new users to initiate their own access to the data from the original and verified source to avoid this risk.

#### Host and phenotype data

This clause addresses concerns for human participants, and particularly that human data associated with microbial data could potentially create a risk to participants through re-identification. The requirements are designed to minimise and/or completely remove this risk to human participants associated with the microbial data. In addition, sequence data from non-human hosts such as agricultural animals, plants or crops should also be carefully managed to ensure that there is not inappropriate sharing of host data that contravenes legislation and agreements, for example the Nagoya Protocol and the Convention on Biological Diversity (www.cbd.int), the World Trade Organisation Agreement on Trade-Related Aspects of Intellectual Property Rights, agreements regulating sharing of animal genetic resources and national legislation relating to plant and animal breeding and protection of varieties.

#### Geospatial data

This clause recognises that geospatial data may create risks to individuals, households and communities if used inappropriately. The requirements outlined here are designed to mitigate this risk while raising awareness of the potential to do harm and create stigma for communities and individuals when creating maps that show the source of microbial data. This clause requires that geospatial heat maps have high enough counts per aggregate data area, and the area covered is sufficiently high that these maps cannot be used to infer the identity of individuals, households or specific communities.

#### Intellectual property (IP)

This clause protects the IP rights of the data generator, setting a default that the IP arising from the use of those data always remains with the data generator unless a valid intellectual property transfer agreement is in place for the secondary use of those data by the specific end user.

#### Opportunity for collaboration

This clause upholds the opportunity for data generators to be involved in the secondary analysis of data they have generated, where this is feasible. This recognises the need to address ongoing inequities where data generators from under-resourced environments may struggle to build analytical capacity if they do not have the opportunity to be involved in downstream analysis of the data they are generating. In addressing this issue, we recognise that it may not always be feasible to include all data generators in every analysis, but we do believe a genuine effort should be made to discuss the potential involvement of the data generator in onward analyses and to extend this invitation to collaborate. At the least, this clause aims to raise awareness about inequitable practices, the need to support global equity in global research activities, and to prompt researchers to consider if they are able to have an opportunity to drive equity and capacity development in their use of data generated by other parties. In addition, as per the example provided earlier, it remains the choice of the data generator to waive this clause if they do not wish to be involved in onward analyses.

To further illustrate how the Accord may assist in addressing some of the existing challenges, grey areas and potential for data-sharing misunderstandings and mis-use, we have also provided a selection of illustrative scenarios in Box 2. While these scenarios are based on our own experiences and observations of real-world examples, we have chosen to compile generalised scenarios as we believe these are more helpful in illustrating potential benefits for researchers in adopting the PHA4GE Microbial Data-Sharing Accord.

Box 2 **Scenarios illustrating potential applications of the PHA4GE Microbial Data-Sharing Accord**

**Scenario 1: attribution of data generators in secondary use research outputs and aggregated datasets**

Data generators submit data to international repositories to assist in a coordinated global response to a disease threat, and the aggregated data allow users to glean insights from a range of local contexts pertaining to disease transmission dynamics. Currently, however, the aggregation of data does not necessarily ensure that there is attribution of contributions from data producers which made the aggregated data analysis possible.^10 21^ Appropriate attribution from contributors of the original data, according to **Clause 1: Attribution** in the Accord, will foster trust and collegial relationships among scientists, can assist data generators to motivate for ongoing funding by demonstrating the usefulness of their data, and will also provide clarity for conditions of further use the data when combined with other datasets. Ensuring attribution of contributors can thus encourage further data contributions and an increased willingness to share. A reference to the terms of the Accord can be included as a statement on the aggregated dashboards, for example, ‘*These data are shared to inform global disease transmission according to the principles of the PHA4GE Microbial Data Sharing Accord’*. Where datasets are compiled from a very large number of contributions from Open data, it may not be feasible due to space limitations to include the listing and citations directly in a submitted manuscript or on the main page of a website. It is, however, feasible to include listings and citations as supplementary data or to provide a link to a list of contributors published online or in an accessible repository such as GitHub or Google Drive, according to FAIR principles.^1^

**Scenario 2: Aligning public health messaging with new research findings**

Sometimes academic research can result in findings that are damaging to existing public health messaging, and the publication of such research without consultation with data providers can cause a lack of trust to develop between public health practitioners and the academic researchers performing the cutting-edge analyses. The following scenario illustrates this friction, and the solutions provided by adhering to the Accord: A research laboratory performs an analysis using sequences and contextual data from three different public health labs. From their results, they propose recommendations that contradict current public health advice. In accordance with **Clause 2: overview of outputs prior to publication of the Accord**, the researchers share their manuscript containing the recommendations with the public health labs. The following represent lessons learnt in different situations.

- The public health labs do not respond, and the researchers proceed with publication having done their due diligence, or
- The public health labs share additional methodological details with the researchers that were not available in public records, which reveal new information about sampling strategies that create biases that were known to the public health labs but were unknown to the researchers. The researchers take this new information into account in their analyses and reframe their recommendations so they support current public health interventions.
- The researchers take this new information into account in their analyses and find that their original findings are still supported. They share this new information with the public health labs, who subsequently update their advice to the public in advance of publication of the research article. The public health labs also inform institutional decision-makers who are then prepared to respond appropriately to questions from the press.

These interactions foster communication between public health and academic research, and improved the quality of data. The interactions can serve as a conduit to integration of new knowledge into public health practice in a way that benefits all concerned, builds trust between the different stakeholders, and creates opportunities for collaboration in parallel with **Clause 7: opportunity for collaboration**.

**Scenario 3: use of geocoded data**

Pathogen surveillance is aided by the generation of geocodes for samples and participants in studies, together with apps and software that can use geocoded sample data to draw maps and generate transmission networks (eg, Dotmapper^22^). These data can, when used appropriately, support rapid and focused public health responses to pathogen outbreaks and spread. For research purposes and public sharing, however, presenting a map of transmission networks, prevalent and incident cases for infectious diseases may cause stigma for affected individuals, neighbourhoods and communities. Detailed mapping of infectious disease has been seen, for example, during the HIV epidemic in sub-Saharan Africa^23^; for multi-drug resistant tuberculosis outbreaks,^24^ and during the emergence of the SARS-CoV-2 across the different regions of the world.^25^ If datasets are shared according to the PHA4GE Microbial Data-Sharing Accord, **Clause 5: geospatial data** of the Accord ensures that communities and individuals from whom samples are collected are protected from geographical identification and potential stigma. In this way, data generators can be confident that communities who have participated in the primary generation of microbial data will not be compromised by the onward use of the datasets.

**Scenario 4: datasets available through public, open platforms and databases**

Many microbial datasets are made available through open or access-controlled databases. While these datasets are already in the public domain, it is still possible to effectively apply the principles enshrined in the PHA4GE Microbial Data-Sharing Accord, should data providers choose to do so by permitting a statement to be made on the platform together with the uploaded dataset. The reference to the terms of the Accord can be included as a statement, for example, ‘*These data are shared according to the principles of the PHA4GE Microbial Data Sharing Accord’*. If there are certain clauses that are not relevant or do not need to be adhered to, this can be added to the statement; for example, ‘*These data are shared according to the principles of the PHA4GE Microbial Data Sharing Accord, with the exception of Clause 7 as no invitation for collaboration is required’*. In addition, an online data-sharing platform or database may choose to adopt the PHA4GE Microbial Data-Sharing Accord as a baseline set of terms for sharing for all the data distributed through their platform, in which case the platform may advertise this through a statement such as ‘*The XXX Database shares datasets according to the principles of the PHA4GE Microbial Data Sharing Accord’*. By adopting the principles of the Accord in this way, even though the data are openly available, the data generator can be confident that the terms of use are specifically and clearly articulated, and that secondary users of their data have agreed to all the clauses in the Accord as stipulated as the conditions of onward use.

**Scenario 5: regulating onward sharing of data**

Depending on time constraints, existing relationships, assumptions, and/or perceived levels of trust, sometimes organisations can receive reports containing data and analytical results that are not specifically marked as confidential. The onward sharing of these types of information to third parties can sometimes have unforeseen and unintended negative impacts, such as violations of privacy, loss of intellectual property (IP) and opportunities, misinterpretation of data and the creation of mistrust. If an organisation receives data and/or reports describing analyses and feels that other groups would benefit from the knowledge, they must first get permission from the data generators to share those materials with others in adherence with **Clause 3: onward sharing of data**. This practice fosters the creation of chains of custody for data, prevents disputes, and enables data generators to retain control over their IP. This scenario also demonstrates how upholding each of the clauses of the Accord can promote upholding others—in this case upholding the requirement that data will not be shared onwards with third parties simultaneously promotes **Clause 1: attribution** and **Clause 6: intellectual property**.

**Scenario 6: reassigning IP rights when sharing data**

Where data have been shared with a minimal or no formal data-sharing agreement, some difficulties may arise if the secondary data use contributes to new knowledge with a commercial value, for example a novel diagnostic or prognostic pathogen biomarker or new therapeutic products. It is possible that there was no anticipation of potential commercial value arising from the secondary use of the data when they were first shared for secondary use. In this instance in the absence of any other binding agreement, if the parties uphold the Accord, IP for the new commercial use would remain with the data generator in recognition of **Clause 6: intellectual property**. While drawing up a data-sharing agreement prior to use, it should be standard practice to negotiate and document an agreement on the assignment of IP that might arise from the data being shared; but in instances where this has not been undertaken there is an unequivocal default assignment of IP that can avoid further conflict and provide a basis for negotiating a fair onward arrangement for sharing or transfer of IP rights according to the interests of both parties.

## Conclusion

We anticipate that this Accord can (1) clarify an agreed set of clauses that are reasonable, unambiguous and generalisable for most microbial data-sharing scenarios; (2) provide a set of simple, clear and reliable boundaries that are universally understood; and (3) provide a baseline of simple clauses, with the option of adding exceptions or additional clauses to the Accord as needed for individual data-sharing conditions or scenarios.

We believe such an Accord can facilitate sharing of microbial data by helping to articulate practical and generally accepted behavioural norms for data sharing in the international community. Having this baseline set of standard principles could save time and effort for data generators by removing the need for them to repeatedly articulate and redraft these baseline common principles for every new data-sharing arrangement they enter into, and to provide clearly articulated, consensus protections against some of the main concerns that result in reluctance to share data. We believe that widespread use and reference to a core consensus set of principles for sharing of microbial data, such as these, may encourage data sharing by providing an accessible and simple tool to remove ambiguity about how data may be used. While this Accord is not intended to replace detailed and individualised Data-Sharing Agreements that are required for specific use cases, it can provide a baseline code of conduct for working with data available through online platforms and other avenues for accessing data available for sharing. In data-sharing scenarios where no legally binding, bilateral data-sharing agreement is drawn up, the Accord can offer a set of baseline requirements that follow consensus norms for collegial, equitable and respectful sharing of microbial data. The Accord provides an immediately accessible and efficient way for data generators to describe how their data may, and may not, be used during secondary data analysis; and provides a clear guideline for expected behaviours for those undertaking secondary data analysis with data generated elsewhere.

We propose that the Accord should reflect extensive consultation with a wide range of stakeholders in the microbial data community, and should reflect a consensus view arising from these consultations. As the PHA4GE Consortium, with input from preliminary stakeholder engagements, we have provided a first draft of what this Accord might look like (Box 1), as a starting place from which to continue to build a consensus document. This first draft is intended to initiate a conversation that can facilitate the creation of a final version that is a true reflection of a consensus from the microbial data-sharing community. To achieve this, we welcome input and commentary from all stakeholders regarding this first draft of the Data-Sharing Accord.

## Supplementary material

10.1136/bmjgh-2024-016474 online supplemental file 1

## Data Availability

Data sharing not applicable as no datasets generated and/or analysed for this study.

## References

[1] Wilkinson MD, Dumontier M, Aalbersberg IJJ (2016). The FAIR Guiding Principles for scientific data management and stewardship. Sci Data.

[2] World Health Organisation (2016). Policy statement on data sharing by the world health organization in the context of public health emergencies.

[3] Chen Z, Azman AS, Chen X (2022). Global landscape of SARS-CoV-2 genomic surveillance and data sharing. Nat Genet.

[4] Austin CC, Bernier A, Bezuidenhout L (2020). Fostering global data sharing: highlighting the recommendations of the Research Data Alliance COVID-19 working group. Wellcome Open Res.

[5] African Academy of Sciences (2021). Policy paper: recommendations for data and biospecimen governance in Africa | the AAS.

[6] The World Medical Association (1964). The wma declaration of helsinki: ethical principles for medical research involving human subjects.

[7] National Commission for the Protection of Human Subjects of Biomedical and Behavioral Research (1979). The belmont report dept of health, educ, and welfare.

[8] Rice TW (2008). The Historical, Ethical, and Legal Background of Human-Subjects Research. Respir Care.

[9] Timme RE, Wolfgang WJ, Balkey M (2020). Optimizing open data to support one health: best practices to ensure interoperability of genomic data from bacterial pathogens. *One Health Outlook*.

[10] Maxmen A (2021). Why some researchers oppose unrestricted sharing of coronavirus genome data. Nature New Biol.

[11] Ling-Hu T, Rios-Guzman E, Lorenzo-Redondo R (2022). Challenges and Opportunities for Global Genomic Surveillance Strategies in the COVID-19 Era. Viruses.

[12] Kaawa-Mafigiri D, Ekusai Sebatta D, Munabi I (2023). Genetic and Genomic Researchers’ Perspectives on Biological Sample Sharing in Collaborative Research in Uganda: A Qualitative Study. J Empir Res Hum Res Ethics.

[13] Evertsz N, Bull S, Pratt B (2023). What constitutes equitable data sharing in global health research? A scoping review of the literature on low-income and middle-income country stakeholders’ perspectives. BMJ Glob Health.

[14] Wei CR, Kamande S, Lang’at GC (2023). Vaccine inequity: a threat to Africa’s recovery from COVID-19. Trop Med Health.

[15] Massinga Loembé M, Nkengasong JN (2021). COVID-19 vaccine access in Africa: Global distribution, vaccine platforms, and challenges ahead. Immunity.

[16] Schwabish J, Feng A (2021). Do No Harm Guide: Applying Equity Awareness in Data Visualization | Urban Institute.

[17] Brownstein JS, Cassa CA, Mandl KD (2006). No place to hide--reverse identification of patients from published maps. N Engl J Med.

[18] Chief Scientist and Science Division (SCI),Research for Health (RFH) (2022). Sharing and reuse of health-related data for research purposes: who policy and implementation guidance.

[19] Milne R, Patch C (2023). Ethical Challenges Associated with Pathogen and Host Genetics in Infectious Disease. New Bioeth.

[20] Halabi S, Wilder R, Gostin LO (2023). Sharing Pathogen Genomic Sequence Data - Toward Effective Pandemic Prevention, Preparedness, and Response. N Engl J Med.

[21] Moodley K, Cengiz N, Domingo A (2022). Ethics and governance challenges related to genomic data sharing in southern Africa: the case of SARS-CoV-2. Lancet Glob Health.

[22] Smith CM, Hayward AC (2016). DotMapper: an open source tool for creating interactive disease point maps. BMC Infect Dis.

[23] Ratmann O, Kagaayi J, Hall M (2020). Quantifying HIV transmission flow between high-prevalence hotspots and surrounding communities: a population-based study in Rakai, Uganda. Lancet HIV.

[24] Faye LM, Hosu MC, Vasaikar S (2023). Spatial Distribution of Drug-Resistant *Mycobacterium tuberculosis* Infections in Rural Eastern Cape Province of South Africa. Pathogens.

[25] Curtis AJ, Ajayakumar J, Curtis J (2022). Spatial Syndromic Surveillance and COVID-19 in the U.S.: Local Cluster Mapping for Pandemic Preparedness. Int J Environ Res Public Health.

