# Supplementary material for: The PHA4GE Microbial Data-Sharing Accord: establishing baseline consensus microbial data-sharing norms to facilitate cross-sectoral collaboration
Source: BMJ Glob Health. 2024 Oct 30;9(10):e016474. doi: 10.1136/bmjgh-2024-016474 (PMC11529761; doi:10.1136/bmjgh-2024-016474)
Supplement: online supplemental file 1 [file bmjgh-9-10-s001.pdf]

CC BY NC ND

Public Health Alliance for Genomic Epidemiology

The PHA4GE Microbial Data Sharing Accord (Version 1.0)

Contributors: Emma Griffiths, Peter van Heusden , Tsaone Tamuhla, Eddie Lulamba, Anja Bedeker, Michelle Nichol, Alan Christoffels and Nicki Tiffin* *on behalf of the PHA4GE Ethics and Data Sharing Working Group*

Definitions

Data generators: The individuals and institutions that generated and/or collected the microbial data.
Data consumers: The individuals and institutions using microbial data for analysis.
Secondary data use: The use of pre-existing data, that was originally collected for a different purpose, to address new research questions or generate additional insights without conducting new primary data collection.

Introduction

Aim: The Accord aims to establish and delineate a set of baseline consensus norms for the sharing of both openly available and private microbial genomic datasets. The Accord aims to provide a single reference document that can provide a common set of standards for data producers and consumers regarding how secondary use of microbial genomic data will be conducted by those adhering to the principles of the Accord.

Scope: The accord presents a consensus standard for ways of using data for secondary analyses that is clearly articulated in one place and is unambiguous. Whilst the Accord allows for additions and amendments for individual data sharing scenarios, it provides a central baseline agreement underpinned by a common consensus understanding of how data may be used for further analyses.

Benefits: The Accord alleviates the requirement to continually recreate standardised clauses in every new data sharing agreement. It provides a commonly understood and accepted baseline agreement for the use of shared microbial genomic data which can be referred to simply as, for example, "*sharing according to the PHA4GE Microbial Data Sharing Accord*".

Clauses Regarding Secondary Use of Microbial Data

1. **Attribution:**

The Methods section of any type of publication using the data will explicitly describe the source of data, with links/citation where appropriate.

The Acknowledgements section of any type of publication using the data will explicitly describe the source of data, with links/citation where appropriate.

- *Where there are very many data generators they may be listed in a supplementary table for a publication, or an online accessible list, e.g. a webpage or github repo, for oral presentations and other media. In these cases, if feasible, a publication may include a list of contributing institutions.*

2. **Overview of outputs prior to their publication:**

An email or alert will be sent to data generators with a confidential copy of a publication to review prior to publication.

A standard two-week window will be provided for data generators to review the publication and raise any serious concerns that create a real risk for the data generators. No response within the two-week window equates to no concerns raised.

- *The duration of this review window may be increased by data generators but should not be decreased without very good cause.*

3. **Onward sharing of data**

There will be no onward sharing of data to third parties unless there is explicit documented agreement from the data generator for onward sharing, or the data are already available for use as unrestricted open data.

4. **Host and phenotype data**

For human hosts, clinical data attached to sequence data will always be anonymised and de-identified such that they cannot be re-linked to individuals under any circumstances.

Genomic data will always be cleaned of contaminating human sequence by data generators prior to sharing, unless specific agreements and ethical clearance is in place for the onward use of these data.

For other species, genomic data will always be cleaned of contaminating host sequence (for example animal and plant sequence data) to ensure compliance with trade and national resource laws and treaties.

5. **Geospatial data**

Heat maps will be used with aggregate data areas to plot geospatial data, to ensure individuals or specific communities cannot be identified.

- *Individual sample geocoordinates representing humans infected with pathogens will not be presented as dots on maps, in order to prevent re-identification and/or stigma for individuals and communities affected by infectious diseases. Heat maps will ensure that counts per aggregate data area and the area covered are sufficiently high that they cannot be used to infer the identity of individuals, households or specific communities.*

6. **Intellectual Property**

Intellectual property does not transfer to the data user unless specifically and explicitly agreed by the data generator and supported by valid IP transfer agreement documentation.

- *Unless there is a valid written agreement documenting a different arrangement, IP remains with the data generator.*

7. **Opportunity for collaboration**

A reasonable attempt must be made by the data consumer to collaborate with the data generator where feasible, except where the data generator has provided an explicit waiver of this requirement.
